# Life History and Multi-Partner Mating: A Novel Explanation for Moral Stigma Against Consensual Non-monogamy

**DOI:** 10.3389/fpsyg.2019.03033

**Published:** 2020-01-21

**Authors:** Justin K. Mogilski, Virginia E. Mitchell, Simon D. Reeve, Sarah H. Donaldson, Sylis C. A. Nicolas, Lisa L. M. Welling

**Affiliations:** ^1^Department of Psychology, University of South Carolina Salkehatchie, Walterboro, SC, United States; ^2^Department of Psychology, Oakland University, Rochester, MI, United States

**Keywords:** life history, consensual non-monogamy, morality, sociosexuality, risk-taking, disease avoidance

## Abstract

Life history theory (LHT) predicts that individuals vary in their sexual, reproductive, parental, familial, and social behavior according to the physical and social challenges imposed upon them throughout development. LHT provides a framework for understanding why non-monogamy may be the target of significant moral condemnation: individuals who habitually form multiple romantic or sexual partnerships may pursue riskier, more competitive interpersonal strategies that strain social cooperation. We compared several indices of life history (i.e., the Mini-K, the High-K Strategy Scale, pubertal timing, sociosexuality, disease avoidance, and risk-taking) between individuals practicing monogamous and consensually non-monogamous (CNM) romantic relationships. Across several measures, CNM individuals reported a faster life history strategy than monogamous individuals, and women in CNM relationships reported earlier pubertal development. CNM individuals also reported more social and ethical risk-taking, less aversion to germs, and greater interest in short-term mating (and less interest in long-term mating) than monogamous individuals. From these data, we discuss a model to explain how moral stigma toward non-monogamy evolved and how these attitudes may be mismatched to the modern environment. Specifically, we argue that the culture of sexual ethics that pervades contemporary CNM communities (e.g., polyamory, swinging) may attenuate risky interpersonal behaviors (e.g., violent intrasexual competition, retributive jealousy, partner/child abandonment, disease transmission) that are relatively more common among those who pursue multi-partner mating.

## Introduction

Consensual non-monogamy (CNM) refers to any romantic relationship wherein people form consensually non-exclusive romantic or sexual partnerships. Those who practice CNM tend to experience greater moral stigma than those within exclusively monogamous romantic relationships. Compared to monogamous individuals, people are more likely to hold negative attitudes and beliefs about (Conley et al., 2013a; Moors et al., 2013; Grunt-Mejer and Campbell, 2016; Burleigh et al., 2017; Thompson et al., 2018), dehumanize (Rodrigues et al., 2018), and socially distance themselves from Balzarini et al. (2018a) individuals within CNM relationships. Compared to CNM relationships, monogamy is presumed to improve sexual frequency and desire, sexual health, relationship satisfaction, experiences of jealousy, and childcare practices (Conley et al., 2013a,c,d) and is widely believed to be morally superior to CNM (Conley et al., 2013a; Matsick et al., 2014; Grunt-Mejer and Campbell, 2016).

Importantly, these perceptions are discordant with actual practices and outcomes of CNM. For example, CNM individuals are presumed to have worse sexual health than monogamous individuals (Conley et al., 2013a) yet report similar or better sexual health practices compared to monogamous individuals (Conley et al., 2012, 2013b; Lehmiller, 2015). Individuals within CNM relationships report unique benefits from forming multiple intimate relationships (see Moors et al., 2017), including diversified need fulfillment (Mitchell et al., 2014; Muise et al., 2019), more frequent social opportunities (Moors et al., 2017), and more fluid sexual expression (Manley et al., 2015). These benefits are associated with relatively greater relationship satisfaction (Rubel and Bogaert, 2015; Levine et al., 2018), particularly when an individual’s personality is matched to their relationship structure (e.g., when someone with a more unrestricted sociosexuality pursues CNM; Rodrigues et al., 2017, 2019). Therefore, it seems that either monogamy or CNM can improve social relations, romantic satisfaction, and mental health when the option to pursue diverse romantic and sexual strategies allows someone to find and fill their niche.

The harm that third-party stigmatization can introduce to the well-being of people in CNM relationships (e.g., Kirkman et al., 2015) highlights the need to explain moral stigma toward these relationship structures. Most current explanations relate stigma to CNM’s defiance of monogamy as a predominant culture practice (i.e., mononormativity; see Emens, 2004). Day (2013) has argued that stigma against CNM is rooted in defense of a committed relationship ideology, which is the assumption that monogamous marriage is the only relationship structure that provides desirable social and relational outcomes, like loyalty, order, and quality childcare (see also Day et al., 2011). Similarly, many authors have adopted a complementary feminist lens which broadly argues that the predominance of monogamy is a sociohistorically enforced standard that has restricted women’s and other social minorities’ agency, sexual expression, capacity to form extended social support networks, and sexual health (Ziegler et al., 2014; Klesse, 2018; Moors, 2019). Collectively, these perspectives have inspired researchers to document and correct misinformation about CNM practices and outcomes with the hope of alleviating the harmful consequences of stigma.

Although identifying and critically evaluating stereotypes can be an effective intervention against prejudice (Hill and Augoustinos, 2001; Hogan and Mallott, 2005; Kulik and Roberson, 2008; Ateah et al., 2011; Hutzler et al., 2016), studying the evolved psychological mechanisms that underlie prejudicial beliefs against CNM may explain their persistence within cultures resistant to these interventions and help frame the underlying moral anxieties and errors in cognition that lead people to justify wrongful discrimination against CNM. Salvatore (2013) argues that identifying the source of stigma against CNM will require a careful understanding of how CNM is devalued relative to monogamy, either via a lack of familiarity with CNM and its outcomes, or via a perceived threat (i.e., that CNM introduces instability or harm to people and their communities). The current study explores the latter possibility that moral aversion stems from the perception that CNM is a threat to well-being. We propose a novel framework for understanding this stigma by assessing the association between CNM and life history strategies.

Life history theory (LHT) is a framework for understanding individual variation in sexual, reproductive, parental, familial, and social behaviors across the lifespan [reviewed in Figueredo et al. (2006); see also Del Giudice et al., 2015]. It predicts that organisms vary adaptively in how they allocate limited time and resources toward growth and reproduction. This variation can be meaningfully divided into two predominant strategies: a slow life history, whereby individuals delay sexual development and reproduction (i.e., invest more in relatively fewer offspring) and a fast life history, in which individuals experience earlier sexual maturity and produce a greater quantity of offspring (i.e., invest less in relatively more offspring). Each strategy prepares an organism to extract value from its environment according to the physical and social challenges that it experiences throughout development. In relatively more stable environments (e.g., high socioseconomic status, low mortality rate), delayed and restricted reproduction allows resources to be channeled into a few offspring likely to survive. In unpredictable environments, accelerated reproduction hedges the risk of investing too deeply into a single child when that investment is unlikely to pay off.

Although recent work criticizes the validity of applying LHT to trait variation within humans (e.g., Nettle and Frankenhuis, 2019; Zietsch and Sidari, 2019), this predictive lens has been useful for studying psychosocial developmental plasticity within underprivileged environments (see Kuzawa and Bragg, 2012). Relative to a slow life history strategy, people with faster life history strategies prefer immediate over delayed rewards (Griskevicius et al., 2011), reproduce earlier (Boothroyd et al., 2013; Hehman and Salmon, 2019), have more casual sex (Dunkel et al., 2015; Salmon et al., 2016), experience earlier sexual debut and report greater sexual risk-taking (James et al., 2012), pursue social status via dominance rather than prestige (Lukaszewski, 2015), score higher on measures of psychopathy (e.g., boldness, aggression, and disinhibition; Međedović, 2018) and dark personality (i.e., impulsivity, antisociality, entitlement/exploitativeness, Machiavellianism, and aggression; McDonald et al., 2012), and are more likely to use psychoactive substances (Richardson et al., 2014). These traits are advantageous in harsh, unpredictable environments to the extent that they help an individual to competitively capitalize on limited resources. That is, if the future is relatively unpredictable, investing effort into immediate rewards may be a more successful survival strategy than long-term investments that pay off more gradually (Pepper and Nettle, 2017; Ellis and Del Giudice, 2019).

To the extent that CNM relationships are promiscuous, multi-partner mating systems, people may assume that CNM individuals practice faster life history strategies relative to those in monogamous relationships. People who pursue a sociosexually unrestricted mating strategy are perceived to have faster life history qualities (i.e., high impulsivity, high aggression, less education, and origination from more desperate ecologies) and are rated as less trustworthy (Moon et al., 2018). These perceptions appear to be at least somewhat accurate. People from more deprived neighborhoods (i.e., a proxy of fast life history) are less generous in a Dictator Game (Nettle et al., 2011). Likewise, regions with high rates of polygyny and socioeconomic inequality have higher rates of male relative to female mortality (Kruger, 2010), suggesting that these communities experience more intense and violent competition between men for access to limited resources. Furthermore, people readily form lay beliefs about others’ life histories, such that those described as originating from desperate ecologies (e.g., low socioeconomic status, high mortality rates, high crime rates) are presumed to have faster life history qualities (Williams et al., 2016). This suggests that people instinctively use sexual behavior to predict others’ personal qualities (e.g., risk propensity, trustworthiness).

This intuition may explain patterns of moral disgust and condemnation of CNM. Individuals experience moral disgust when a social violation threatens to depose a moral rule that personally benefits them (see Tybur et al., 2013). For example, someone’s preference for long-term mating (see Gangestad and Simpson, 2000) predicts their endorsement of moral rules that constrain others’ sexual behaviors (Weeden et al., 2008; Kurzban et al., 2010). People then condemn others’ behaviors to strategically rally public support for social policies that they perceive to be beneficial (DeScioli and Kurzban, 2009, 2013). People may condemn CNM because they believe that it enables fast life history behaviors (i.e., risk taking, interpersonal antagonism), and that endorsing it will destabilize social unity and cooperation. That is, those who wish to preserve stable, cohesive communities may condemn CNM insofar as sexual promiscuity is indicative of faster life history traits that produce intra-group conflict (e.g., aggressive competition over mates; partner retribution).

People may generally assume that individuals within CNM relationships possess faster life history traits, but it is currently unknown whether these perceptions are accurate. People in CNM relationships consistently report a more unrestricted sociosexuality (Morrison et al., 2013; Rodrigues et al., 2016, 2017, 2019; Mogilski et al., 2017, 2019; Balzarini et al., 2018b). However, it is possible that they do not possess other fast life history qualities that interfere with the long-term, cooperative values endorsed by slow life history strategists. Certainly, the defining quality of CNM relationships is that practitioners are expected to follow strict ethical guidelines that reduce sources of suffering common to multi-partner mating systems (e.g., jealous anxiety, STI transmission, competitive aggression, partner or child abandonment; Hardy and Easton, 2017). Because of the association between unrestricted sociosexual behavior and faster, riskier, competitive interpersonal behaviors, those who stigmatize these relationships may assume that unrestricted sociosexuality is a good predictor of immoral behavior and unethical decision-making.

This study assessed whether life history varies between individuals within monogamous and consensually non-monogamous romantic relationships. We examined life history variation among three groups: (1) those who are currently romantically involved exclusively with only one other person (i.e., monogamous), (2) those non-exclusively involved with only one other person (i.e., open relationship), and (3) those non-exclusively involved with more than one other person (i.e., multi-partner relationships). We measured life history using two self-report measures: the Mini-K (Figueredo et al., 2006) and the High-K Strategy Scale (HKSS; Giosan, 2006), and several complementary measures including self-reported pubertal development (Petersen et al., 1988), sociosexuality [i.e., the Multi-dimensional Model of Sociosexual Orientation (MMSO); Jackson and Kirkpatrick, 2007], the Domain-Specific Risk-Taking Scale (DOSPERT; Blais and Weber, 2006), and the Perceived Vulnerability To Disease Scale (PVDS; Duncan et al., 2009).

Overall, we predicted that individuals within monogamous relationships would report a slower life history than those within open and multi-partner CNM relationships. We expected aspects of life history that correspond to greater sexual promiscuity (e.g., unrestricted sociosexuality, earlier pubertal development) would be higher among open and CNM participants than among those in monogamous relationships. However, to the extent that those within CNM relationships follow popular guidelines that prevent interpersonal exploitation and dangerous sexual practices (Hardy and Easton, 2017), we predicted that they would not be more willing to exploit others for personal gain or to expose themselves to pathogens (e.g., STIs) than monogamous individuals. We expected risk-taking, and in particular facets of risk-taking that expose others to danger (i.e., ethical and health risk-taking) and perceived vulnerability to disease to be no different than those in monogamous relationships. Furthermore, we used the MMSO to measure sociosexuality because it measures interest in short- and long-term relationships on separate continua. Compared to the SOI-R, which has already been used extensively in prior CNM research, this will permit us to not only examine whether CNM individuals are more interested in casual sex than monogamous individuals, but also whether they are relatively less interested in long-term, committed relationships. Although the former has been well-documented (Morrison et al., 2013; Rodrigues et al., 2016, 2017, 2019; Mogilski et al., 2017, 2019; Balzarini et al., 2018b), the latter has not. A person could theoretically be high or low on either or both measures.

## Materials and Methods

### Ethics Statement

This study was carried out in accordance with the recommendations of and approval by the Oakland University Institutional Review Board. All subjects gave written informed consent in accordance with the Declaration of Helsinki.

### Participants

Participants were recruited from social media (e.g., Facebook, Reddit, Twitter) and an undergraduate college population as part of a three-phase online study, whereby each phase tested a different research question related to CNM. A distinct sample of participants was collected for each phase, but participants did have the option of participating in other phases. Data for this study are exclusively from phase 1 of this project. For this phase, we collected a total of 923 responses. To identify whether respondents completed the survey more than once, participants provided anonymous identifiers by indicating (1) the letter of their middle name, (2) the first letter of their mother’s first name, (3) the first letter of their sex, (4) the number of the month they were born, and (5) the first letter of their ethnic background. Duplicate participant entries were deleted (*n* = 40) if two or more sets of IP addresses and responses to the anonymous identifier items matched. Participants who completed the survey in <15 min (*n* = 85) were excluded. Participants who indicated they were in an exclusive relationship but were currently involved with more than one person (i.e., non-consensual non-monogamy; *n* = 5) and those who provided inconsistent relationship information (e.g., reported that they were currently involved with only one partner but also indicated 2+ current partners; *n* = 10) were also excluded from analyses.

The final sample consisted of 783 participants (age: *M* = 23.49 years, *SD* = 7.91, range 18–77 years). Participants resided in the United States (91.6%), Europe (4.5%), Oceania (1.9%), Canada (1.7%), Asia (0.1%), South America (0.1%), and Africa (0.1%). Approximately 70% were from Michigan. Participants were asked to identify their biological sex as either male (*n* = 183) or female (*n* = 600), but were also asked to identify their gender (male = 184; female = 579; “other” = 20). Other gendered individuals identified as genderfluid or genderqueer (*n* = 12), agender (*n* = 3), non-binary (*n* = 2), semi-androgynous (*n* = 1), or did not provide a gender identity (*n* = 2). Our sample also contained four transmen and two transwomen. Because our sample of trans and other-gendered individuals was not large enough to conduct separate analyses, biological sex was used for comparisons of sex differences. Participants identified as White (82.6%), Black (6.5%), Asian (4.8%), Hispanic/Latino (1.9%), or Other (4.2%), and reported their sexual orientation as heterosexual (73.8%), bisexual or pansexual (23.2%), homosexual (1.8%), or asexual (0.9%).

All participants reported currently being in a romantic relationship of some type. Following previous methods (Mogilski et al., 2017, 2019), two criteria were used to distinguish between individuals in monogamous, open, and CNM relationships. First, participants reported whether their romantic relationship was exclusive (i.e., you and your partner agree to not date other people) or non-exclusive (i.e., you and your partner(s) agree that dating other people is permitted) and whether they were currently in a romantic and/or physical relationship with only one person or with more than one person. Participants who reported being in an exclusive romantic or physical relationship with only one person were classified as “monogamous.” Those who reported being in a non-exclusive romantic or physical relationship with more than one person were classified as “multi-partner.” Those who reported being in a non-exclusive romantic or physical relationship with only one person were classified into a third group called “open relationship.”

Using these criteria, the sample consisted of 538 monogamous (416 women; age: *M* = 20.65 years, *SD* = 4.70, range = 18–71 years; sexual orientation: 90.0% heterosexual, 8.2% bisexual/pansexual, 1.7% homosexual, 0.9% asexual), 149 multi-partner (117 women; age: *M* = 31.67 years, *SD* = 9.44, range = 18–58 years; sexual orientation: 28.2% heterosexual, 70.5% bisexual/pansexual, 0.7% asexual), and 96 open relationship (67 women; age: *M* = 26.8 years, *SD* = 9.55, range = 18–77 years; sexual orientation: 54.2% heterosexual, 44.8% bisexual/pansexual, 5.2% homosexual, asexual 1.0%) participants. Multi-partner participants reported their current number of partners (48.3% two partners, 26.2% three partners, 20.9% four or more partners), and described their romantic relationships using one or more of the following descriptors:

(1) “I am in a primary relationship with one person (i.e., an emotional/sexual relationship characterized by a high degree of commitment, shared life goals, and affection) and in secondary relationships with one or more other people (i.e., close, ongoing emotional/sexual relationship(s), but with a lesser degree of commitment than a primary relationship)” (*n* = 93).
(2) “I am equally involved with only two people” (*n* = 31).
(3) “I am equally involved with more than two people” (*n* = 13).
(4) “I am involved in a poly ‘web’, ‘family’, or ‘intimate network’ (i.e., a social web resulting from having romantic relationships among you, your romantic partners, their romantic partners, and so forth)” (*n* = 46).

Monogamous and open participants did not report involvement in any of these relationship structures.

### Materials and Procedures

All measures were presented using the online survey program Qualtrics. The order in which participants completed each set of measures was randomized across and counterbalanced within tasks. Informed consent was obtained from all individual participants included in the study. After providing informed consent, participants answered questions about themselves, including a demographic questionnaire (age, gender, race/ethnicity, and sexual orientation), and completed self-report measures of life history, pubertal development, sociosexual orientation, perceived vulnerability to disease, and risk-taking.

#### Life History

We measured overall life history strategy using the Mini-K (Figueredo et al., 2006; 20 items; α = 0.758) and the HKSS (Giosan, 2006; 22-items; α = 0.842). The Mini-K assesses several domains of social and sexual behavior, including an individual’s contact with and support from family, friends, and community (e.g., “I often get emotional support and practical help from my friends/community”); their relationship quality with biological relatives (e.g., “while growing up, I had a close and warm relationship with my biological mother/father”); their capacity for insight, planning, and self-control (e.g., “I often make plans in advance”); and their preference for intimacy and sex with multiple romantic partners (e.g., “I would rather have one than several sexual relationships at a time”; anchors: 1 = strongly disagree, 7 = strongly agree). The HKSS similar assesses life history qualities such as health and attractiveness (e.g., “I don’t have major medical problems,” “I am in good physical shape”), upward social mobility (e.g., “My training and experience are likely to bring me opportunities for promotion and increased income in the future”), social capital (e.g., “If something bad happened to me, I’d have many friends ready to help me”), and risk avoidance (e.g., “I live in a comfortable and secure home”; anchors: 1 = strongly disagree, 5 = strongly agree). Higher average scores on both measures indicate a slower life history strategy.

#### Sociosexual Orientation

Two attitudinal aspects of sociosexual orientation were measured using items from the MMSO (Jackson and Kirkpatrick, 2007; α = 0.643): (1) short-term mating orientation (STMO; 10 items, e.g., “I can easily imagine myself being comfortable and enjoying ‘casual’ sex with different partners”) and (2) long-term mating orientation (LTMO; seven items, e.g., “I am interested in maintaining a long-term relationship with someone special”; anchors: 1 = strongly disagree, 7 = strongly agree). Higher average scores on both measures indicate greater preference.

#### Pubertal Development

Pubertal timing was measured by asking participants to recall whether they had experienced pubertal events relatively earlier or later than their same-sex peers (e.g., changes in voice pitch, facial skin clarity, body hair development; Petersen et al., 1988). Some items were different for men and women depending on their sex-specificity (e.g., “do you think you started having wet dreams earlier or later than your peers?,” “do you think your first period was any earlier or later than most other girls?”; anchors: 1 = much earlier, 5 = much later). Participants could report “I don’t know.” These responses were excluded from analyses. Higher average scores indicate delayed sexual maturation.

#### Perceived Vulnerability to Disease

Chronic concerns about susceptibility to infectious disease transmission were assessed using the PVDS (Duncan et al., 2009; 15 items) which measures two domains: perceived infectability (e.g., “I am more likely than the people around me to catch an infectious disease”; α = 0.85) and germ aversion (e.g., “I dislike wearing used clothes because you do not know what the last person who wore it was like”; α = 0.77; anchors: 1 = strongly disagree, 7 = strongly agree). Higher average scores indicate greater perceived vulnerability.

#### Risk-Taking

Attitudes to various risk-taking behaviors were measured using the *Domain Specific Risk-Taking* (DOSPERT; Blais and Weber, 2006; 30 items; α = 0.824) questionnaire. This questionnaire evaluates how likely a participant believes they are to take risks across five domains: *Ethical* (e.g., “passing off somebody else’s work as your own”; α = 0.64), *Financial* (e.g., “bettering a day’s income at a high-stake poker game”; α = 0.75), *Health/Safety* (e.g., “engaging in unprotected sex”; α = 0.58), *Recreational* (e.g., “going down a ski run that is beyond your ability”; α = 0.79), and *Social* (e.g., “speaking your mind about an unpopular issue in a meeting at work”; α = 0.66; anchors: 1 = extremely unlikely, 7 = extremely likely). Higher average scores indicate greater risk-taking propensity.

## Results

All *post hoc* tests were Bonferroni corrected. Their adjusted *p*-values are reported. Because monogamous, open, and multi-partner participants significantly differed by age, *F*(2,774) = 176.09, *p* < 0.001, ${\text{η}}_{\text{p}}^{2}$ = 0.31), we ran our analyses both including and excluding age as a covariate. Patterns of significance were the same in both sets of analyses. Analyses excluding age as a covariate are reported.

### Life History Measures

#### Mini-K

A 2 (participant sex) × 3 (relationship type) between-subjects ANOVA compared scores on the Mini-K among women and men within each relationship type. There was a main effect of participant sex, *F*(1,777) = 12.98, *p* < 0.001, ${\text{η}}_{\text{p}}^{2}$ = 0.02, such that men reported lower scores (i.e., a faster life history strategy; *M* = 4.99, *SD* = 0.72) than women (*M* = 5.26, *SD* = 0.69), and a main effect of relationship type, *F*(2,777) = 32.67, *p* < 0.001, ${\text{η}}_{\text{p}}^{2}$ = 0.08. Participants in multi-partner (*M* = 4.81, *SD* = 0.58, *p* < 0.001) and open (*M* = 4.93, *SD* = 0.80, *p* < 0.001) relationships reported lower scores than monogamous participants (*M* = 5.36, *SD* = 0.66). There was no significant difference between those in multi-partner and open relationships (*p* = 0.539) and no significant interaction, *F*(2,777) = 0.05, *p* = 0.949.

#### HKSS

A 2 (participant sex) × 3 (relationship type) between-subjects ANOVA compared scores on the HKSS among men and women within each relationship type. There was a main effect of relationship type, *F*(2,777) = 4.67, *p* = 0.010, ${\text{η}}_{\text{p}}^{2}$ = 0.01, such that people in open relationships scored lower (i.e., a faster life history strategy; *M* = 3.81, *SD* = 0.53) than those in monogamous (*M* = 3.97, *SD* = 0.46, *p* = 0.007), but not multi-partner (*M* = 3.92, *SD* = 0.40, *p* = 0.124), relationships. There was a marginally significant main effect of sex, *F*(1,777) = 3.59, *p* = 0.059, ${\text{η}}_{\text{p}}^{2}$ = 0.01, such that men (*M* = 3.87, *SD* = 0.47) scored lower than women (*M* = 3.96, *SD* = 0.46). There was no significant interaction, *F*(4,777) = 0.99, *p* = 0.372.

To assess the construct validity of the Mini-K and HKSS, bivariate correlations were calculated among these scores and the pubertal development, MMSO, PVDS, and DOSPERT scores (Table 1).

**TABLE 1:** Bivariate correlations among measures of life history (Mini-K and HKSS), male and female pubertal development, risk-taking (DOSPERT), disease avoidance (PVDS), and long- and short-term mating orientations (MMSO).

	HKSS	Mini-K	Male PD	Female PD	MMSO Sh.	MMSO L.
1. HKSS		0.586^∗∗^	–0.096	0.014	–0.154^∗∗^	0.186^∗∗^
2. Mini-K	0.586^∗∗^		–0.001	0.023	–0.460^∗∗^	0.344^∗∗^
3. Male pubertal development	–0.096	–0.001			–0.058	0.089
4. Female pubertal development	0.014	0.023			−0.088^∗^	0.045
5. MMSO short-term	–0.154^∗∗^	–0.460^∗∗^	–0.058	−0.088^∗^		–0.324^∗∗^
6. MMSO long-term	0.186^∗∗^	0.344^∗∗^	0.089	0.045	–0.324^∗∗^	
7. PVDS perceived infectability	–0.164^∗∗^	–0.011	0.019	–0.027	–0.045	–0.026
8. PVDS germ aversion	0.107^∗∗^	0.315^∗∗^	–0.075	0.029	–0.377^∗∗^	0.213^∗∗^
9. DOSPERT social	–0.074^∗∗^	−0.261^∗^	0.036	–0.058	0.371^∗∗^	–0.168^∗∗^
10. DOSPERT recreational	0.180^∗∗^	0.034	−0.148^∗^	–0.012	0.107^∗∗^	0.082^∗^
11. DOSPERT financial	0.035	–0.004	–0.039	0.018	0.176^∗∗^	–0.045
12. DOSPERT health/safety	–0.059	–0.187^∗∗^	–0.098	–0.011	0.336^∗^	–0.028
13. DOSPERT ethical	–0.182^∗∗^	–0.259^∗∗^	0.008	0.036	0.426^∗∗^	–0.155^∗∗^

	**PVDS Inf.**	**PVDS Ger.**	**Social**	**Recreat.**	**Finan.**	**Heal./Safe.**	**Ethical**

1. HKSS	–0.164^∗∗^	0.107^∗∗^	−0.074^∗^	0.180^∗∗^	0.035	–0.059	–0.182^∗∗^
2. Mini-K	0.011	0.315^∗∗^	–0.261^∗∗^	0.034	–0.004	–0.187^∗∗^	–0.259^∗∗^
3. Male pubertal development	0.019	–0.075	0.036	−0.148^∗^	–0.039	–0.098	0.008
4. Female pubertal development	–0.027	0.029	–0.058	–0.012	0.018	–0.011	0.036
5. MMSO short-term	–0.045	–0.377^∗∗^	0.371^∗∗^	0.107^∗∗^	0.176^∗∗^	0.336^∗∗^	0.426^∗∗^
6. MMSO long-term	–0.026	0.213^∗∗^	–0.168^∗∗^	0.082^∗^	–0.045	–0.028	–0.155^∗∗^
7. PVDS perceived infectability		0.222^∗^	–0.017	–0.095^∗∗^	–0.032	–0.028	0.032
8. PVDS germ aversion	0.222^∗∗^		–0.273^∗∗^	–0.123^∗∗^	–0.009	–0.232^∗∗^	–0.173^∗∗^
9. DOSPERT social	–0.017	–0.273^∗∗^		0.146^∗∗^	0.176^∗∗^	0.228^∗∗^	0.206^∗^
10. DOSPERT recreational	–0.095^∗∗^	–0.123^∗∗^	0.146^∗∗^		0.280^∗∗^	0.391^∗∗^	0.158^∗∗^
11. DOSPERT financial	–0.032	–0.009	0.176^∗∗^	0.280^∗∗^		0.275^∗∗^	0.404^∗∗^
12. DOSPERT health/safety	–0.028	–0.232^∗∗^	0.228^∗∗^	0.391^∗∗^	0.275^∗∗^		0.482^∗∗^
13. DOSPERT ethical	0.032	–0.173^∗∗^	0.206^∗∗^	0.158^∗∗^	0.404^∗∗^	0.482^∗∗^	

*^∗∗^*p* < 0.01. ^∗^*p* < 0.05.*

### MMSO

Two 2 (participant sex) × 3 (relationship type) between-subjects ANOVA compared STMO and LTMO scores on the MMSO among men and women within each relationship type. For STMO, there was a main effect of sex, *F*(1,767) = 32.44, *p* < 0.001, ${\text{η}}_{\text{p}}^{2}$ = 0.04, such that men (*M* = 3.96, *SD* = 1.10) scored higher than women (*M* = 3.16, *SD* = 1.27). There was also a main effect of relationship type, *F*(2,767) = 67.38, *p* < 0.001, ${\text{η}}_{\text{p}}^{2}$ = 0.15, such that multi-partner individuals scored higher (*M* = 4.45, *SD* = 0.86) than both open individuals (*M* = 3.87, *SD* = 1.33, *p* = 0.003) and monogamous individuals (*M* = 2.95, *SD* = 1.19, *p* < 0.001). Open participants also scored significantly higher than monogamous participants (*p* < 0.001). There was no significant interaction, *F*(2,767) = 2.56, *p* = 0.078, ${\text{η}}_{\text{p}}^{2}$ = 0.01.

For LTMO, there was a main effect for relationship type, *F*(2,768) = 52.81, *p* < 0.001, ${\text{η}}_{\text{p}}^{2}$ = 0.12, such that monogamous individuals scored higher on LTMO (*M* = 5.17, *SD* = 0.40) than those in open (*M* = 4.96, *SD* = 0.51, *p* < 0.001) and multi-partner relationships (*M* = 4.61, *SD* = 0.61, *p* < 0.001). There was also a significant difference between open and multi-partner individuals (*p* < 0.001). There was no main effect of sex, *F*(2,768) = 0.90, *p* = 0.343, nor a significant interaction, *F*(2,768) = 1.86, *p* = 0.156.

### Self-Reported Pubertal Timing

Two three-way between-subjects ANOVA compared male and female pubertal timing measures across monogamous, open, and multi-partner relationships. There was a main effect of relationship type for women, *F*(2,596) = 5.23, *p* = 0.006, ${\text{η}}_{\text{p}}^{2}$ = 0.02, but not for men, *F*(2,179) = 1.80, *p* = 0.168, ${\text{η}}_{\text{p}}^{2}$ = 0.02. Women in multi-partner relationships reported earlier pubertal development relative to their peers (*M* = 2.79, *SD* = 0.69) than did women in monogamous relationships (*M* = 3.02, *SD* = 0.69, *p* = 0.004). Women in open relationships (*M* = 2.99, *SD* = 0.70) were not significantly different from women in multi-partner and monogamous relationships.

### PVDS

A 2 (participant sex) × 3 (relationship type) between-subjects MANOVA compared scores on the two domains of the PVDS (i.e., perceived infectability and germ aversion) among men and women within each relationship type. For perceived infectability, there was a main effect of sex, *F*(1,776) = 21.39, *p* < 0.001, ${\text{η}}_{\text{p}}^{2}$ = 0.03, such that women perceived themselves as more susceptible to infection (*M* = 3.68, *SD* = 1.40) than did men (*M* = 2.99, *SD* = 1.10). However, there was no main effect of relationship type, *F*(2,776) = 2.00, *p* = 0.136, nor a significant interaction, *F*(2,776) = 2.35, *p* = 0.096.

For germ aversion, there was a main effect of sex, *F*(1,776) = 4.91, *p* = 0.027, ${\text{η}}_{\text{p}}^{2}$ = 0.01. Women reported greater aversion to germs (*M* = 3.96, *SD* = 1.16) than men (*M* = 3.56, *SD* = 1.08). There was also a main effect of relationship type, *F*(2,776) = 27.62, *p* < 0.001, ${\text{η}}_{\text{p}}^{2}$ = 0.07. People in monogamous relationships reported greater aversion to germs (*M* = 4.13, *SD* = 1.11) than multi-partner (*M* = 3.17, *SD* = 1.02, *p* < 0.001) and open (*M* = 3.46, *SD* = 1.04, *p* < 0.001) individuals. Multi-partner and open individuals were not significantly different from each other (*p* = 0.468). There was also a significant interaction, *F*(2,776) = 3.04, *p* = 0.048, ${\text{η}}_{\text{p}}^{2}$ = 0.01. Women in monogamous relationships were more germ averse (*M* = 4.25, *SD* = 1.08) than men in monogamous relationships (*M* = 3.72, *SD* = 1.11), *t*(536) = −4.74, *p* < 0.001, but there were no sex differences within open (women: *M* = 3.28, *SD* = 0.95; men: *M* = 3.54, *SD* = 1.07), *t*(94) = −1.16, *p* = 0.249, or multi-partner (women: *M* = 3.21, *SD* = 0.97; men: *M* = 3.16, *SD* = 1.04), *t*(146) = 0.24, *p* = 0.815, relationships.

### DOSPERT

A 2 (participant sex) × 3 (relationship type) between-subjects MANOVA compared scores on the five domains of the DOSPERT among men and women within each relationship type. For each domain, there was main effect of sex (all *F*s > 9.38) such that men scored higher than women on social (*M* = 5.12, *SD* = 1.01; *M* = 4.79, *SD* = 1.02, *p* = 0.001), recreational (*M* = 3.94, *SD* = 1.41; *M* = 3.45, *SD* = 1.40, *p* = 0.002), financial (*M* = 2.75, *SD* = 1.08; *M* = 2.29, *SD* = 0.93, *p* < 0.001), health/safety (*M* = 3.42, *SD* = 1.12; *M* = 2.99, *SD* = 1.08, *p* < 0.001), and ethical risk-taking (*M* = 2.57, *SD* = 1.07; *M* = 2.16, *SD* = 0.83, *p* < 0.001).

There were two main effects for relationship type: social risk-taking, *F*(2,776) = 61.68, *p* < 0.001, ${\text{η}}_{\text{p}}^{2}$ = 0.14, and ethical risk-taking, *F*(2,776) = 8.08, *p* < 0.001, ${\text{η}}_{\text{p}}^{2}$ = 0.02. People in multi-partner (*M* = 5.56, *SD* = 0.87, *p* < 0.001) and open (*M* = 5.35, *SD* = 0.98, *p* < 0.001) relationships scored higher on social risk-taking than monogamous individuals (*M* = 4.59, *SD* = 0.94). Those in open relationships were not significantly different from multi-partner individuals (*p* = 0.299). Likewise, people in multi-partner (*M* = 2.51, *SD* = 0.89, *p* = 0.007) and open (*M* = 2.43, *SD* = 1.02, *p* = 0.005) relationships scored higher on ethical risk-taking than did monogamous people (*M* = 2.15, *SD* = 0.87), and there were no differences between multi-partner and open individuals (*p* = 1.00).

Finally, there was a significant interaction for ethical risk-taking, *F*(2,776) = 3.40, *p* = 0.034, ${\text{η}}_{\text{p}}^{2}$ = 0.01. Men scored significantly higher than women on ethical risk-taking within monogamous, *t*(536) = 4.28, *p* < 0.001, and open relationships, *t*(94) = 3.88, *p* < 0.001, but not multi-partner relationships, *t*(146) = 0.84, *p* = 0.403. All other main effects and interactions were not significant (all *F*s < 2.67, all *p*s > 0.069).

## Discussion

We compared self-report indices of life history across men and women within monogamous, open, and multi-partner romantic relationships. Collectively, our results suggest that pursuit of CNM is associated with a faster life history strategy. Individuals within open and multi-partner relationships reported lower scores (i.e., a faster life history) on the Mini-K than those in monogamous relationships. Open individuals also reported lower scores on the HKSS than both monogamous and multi-partner individuals, who were no different from one another.

That individuals within CNM relationships report a faster life history makes sense in light of previous research on the association between faster life histories and promiscuous mating systems. CNM individuals’ preference for multiple sexual and romantic partners has been documented across several samples (Morrison et al., 2013; Rodrigues et al., 2016, 2017, 2019; Mogilski et al., 2017, 2019; Balzarini et al., 2018b) and is replicated again in this study using an alternative measure of sociosexuality (i.e., the MMSO) that separately measures affinity toward short- and long-term partnerships. We found that those in multi-partner relationships reported a more STMO than those in open and monogamous relationships, and open individuals reported a more STMO than monogamous people. Interestingly, those in multi-partner relationships also reported less interest in long-term committed romantic relationships than monogamous, but not open, individuals. It is possible that CNM individuals, and particularly those that maintain several concurrent romantic relationships, form fewer enduring partnerships than those in monogamous relationships. However, this is not consistent with prior research. Séguin et al. (2017) found that individuals within polyamorous relationships reported longer relationships than those in monogamous and open relationships, and all three relationship types reported similar levels of partner commitment. Similarly, Mogilski et al. (2017) compared relationship length between monogamous and CNM individuals’ primary and secondary relationships. Although they found that monogamous relationships tended to be older than secondary relationships, CNM primary relationships tended to be older than monogamous relationships. This suggests that those in CNM relationships regularly form long-term enduring relationships but are perhaps selective about with whom they maintain those relationships. That is, people who form multi-partner relationships may desire and actively seek a variety of intimate partners, but only maintain partnerships if they are of high quality. Balzarini et al. (2017) reported that primary partnerships tend to entail more commitment than secondary partnerships, and Mitchell et al. (2014) likewise found that polyamorous individuals report greater commitment to one partner than the other. Alternatively, LTMO may differ across different types of CNM. We did not collect data to distinguish different types of multi-partner relationships, but individuals interested in polyamory (i.e., multiple emotionally intimate relationships) may be more oriented toward long-term relationships than those interested in exclusively sexual extradyadic relationships.

Our complementary findings suggest that life history differences between monogamous and CNM individuals extend beyond sociosexuality. Women within multi-partner, but not open, relationships reported earlier sexual debut than women within monogamous relationships. There were no differences in self-reported pubertal timing among men. This is consistent with research showing that early sexual maturity is associated with a faster life history in women (Byrd-Craven et al., 2007; James et al., 2012; also see Hehman and Salmon, 2019), particularly within western industrialized societies (Sear et al., 2019). Scores on the PVDS also revealed that individuals within CNM and monogamous relationships did not differ in their perceived infectability. However, monogamous individuals reported greater germ aversion than both multi-partner and open individuals, while the latter were equally averse. This is consistent with work showing that those who score higher on the Mini-K (i.e., slow life history) report greater pathogen, sexual, and moral disgust than those who score lower (Frederick et al., 2018). For slow strategists, this aversion may motivate protective avoidance of risks that threaten long-term well-being. For fast strategists, a higher threshold for disgust would allow them to capitalize on opportunities despite possible risks (e.g., exposure to disease, interpersonal exploitation). However, these individuals may likewise fail to avoid sexual disease risk, which may become a community health issue. Finally, we also observed that those in multi-partner and open relationships scored higher than monogamous people on social and ethical (though not health) risk-taking. This suggests that CNM individuals may be more likely to disregard how their behaviors are perceived by or affect the well-being of others, but supports research showing that those in CNM relationships tend to be conscientious about sexual health (Conley et al., 2012, 2013b). Collectively, these findings suggest that differences in life history between monogamous and CNM individuals do not merely reflect differences in sociosexuality. Rather, people who are interested in pursuing a CNM relationship may be predisposed to a faster life history strategy.

### CNM, Morality, and Sexual Ethics

Knee-jerk condemnation of CNM can produce wrongful discrimination that harms personal and community well-being. For instance, those in CNM relationships typically report being more secretive about their non-primary (or pseudo-non-primary) partners (Balzarini et al., 2019), presumably to avoid third-party punishment. Indeed, Conley et al. (2012) found that women who fear condemnation are less willing to accept an offer of casual sex that they would otherwise enjoy pursuing. This fear of judgment can cause anxiety that prevents those who practice CNM from seeking sexual health services (e.g., STD testing), particularly within rural communities where reputation can be more easily tracked (Kirkman et al., 2015). Moreover, therapists and clinicians who assume that monogamy is a universal relationship ideal may inadvertently marginalize or mistreat patients who are oriented toward multi-partner mating (see Finn et al., 2012; Brandon, 2016; van Tol, 2017; Cassidy and Wong, 2018). In fact, Schechinger et al. (2018) found that CNM individuals reported that therapy was more helpful when therapists were more affirmative about their relationship structure (e.g., did not make an issue of their relationship structure when it was not relevant).

It is possible that moral stigma toward CNM (see Moors et al., 2013) stems from aversion to the high-risk, competitive interpersonal strategies that are characteristic of a fast life history (see Wang et al., 2009; Figueredo and Jacobs, 2010; Kruger, 2010; Griskevicius et al., 2011). Commitment to a faster life history strategy can lead to greater risk-taking (Hampson et al., 2016; Mishra et al., 2017), impulsivity (Frankenhuis et al., 2016; Maner et al., 2017), and aggression against others (Figueredo et al., 2018). Also, robust indicators of faster life history, such as paternal absenteeism and adolescent fertility, predict national rates of criminal violence (Minkov and Beaver, 2016), child maltreatment, and homicide (Hackman and Hruschka, 2013). Moral condemnation of multi-partner mating may thereby occur when condemners believe that monogamy prevents competitive contests for mates, enhancing cooperation within groups and reducing negative physical and mental health outcomes. In other words, though fast life history traits can help individuals cope with an unpredictable environment (Figueredo and Jacobs, 2010; Frankenhuis et al., 2016; Young et al., 2018), they may conflict with the optimal social strategy pursued by slow life history strategists. Baumard and Chevallier (2015) argue that fast life history behaviors may be moralized to the extent that slow strategists promote cooperation, self-regulation, and restricted sociosexuality, and condemn “fast” behaviors such as selfishness, conspicuous sexuality, and materialism. By espousing moral values that promote delayed gratification, sexual monogamy, and altruism, slow life history strategists may condemn multi-partner mating to create stable, cohesive communities that invest in long-term reciprocity and extended prosociality.

Although our data support the conclusion that CNM is associated with fast life history traits, it is important to note that our study assesses dispositional tendencies and not how these tendencies are modified by cultural practices within the CNM community. People who prefer multi-partner mating may have a proclivity toward pursuing a faster life history, but most modern CNM communities have well-developed guidelines for pursuing multi-partner relationships safely and ethically (see Anapol, 1997; Wosick-Correa, 2010; Deri, 2015; Hardy and Easton, 2017). Sexual ethics within CNM communities, including effective birth control methods, may help manage and diminish the traditional costs of competitive, high-risk, promiscuous mating environments. CNM individuals take precautions to attenuate distress caused by a partner’s extradyadic involvement (Jackson and Scott, 2004; McLean, 2004; Visser and McDonald, 2007). Those in CNM relationships are just as (or more) likely to practice safe sexual practices than people in monogamous relationships (Conley et al., 2012, 2013b; Lehmiller, 2015). They are also expected to practice open communication, honesty, emotional intimacy, and consent-seeking to reduce the threat of partner defection or resource diversion. Scoats and Anderson (2019) interviewed men and women who engaged in mixed-sex threesomes and found that open communication reduced feelings of exclusion. Similarly, Aguilar (2013) studied two communal living groups practicing polyamory and reported that both cultures discouraged aggression and competition among males within the community.

By reducing the social anxiety that accompanies multi-partner competition, individuals within CNM relationships may experience relationship and health outcomes on par with (or better than) those who pursue monogamy. Those within multi-partner relationships that include ethical treatment of and consent among partners typically experience more positive relationship and health outcomes than those who pursue non-consensual non-monogamy (i.e., adultery; Levine et al., 2018). Compared to those in monogamous relationships, CNM individuals report experiencing less emotional jealousy (Mogilski et al., 2019), and spend less time actively trying to retain their mate (Mogilski et al., 2017, 2019), which may alleviate conflict in relationships where one or both partners desire extradyadic intimacy. Indeed, people with an unrestricted sociosexuality report greater satisfaction within CNM relationships than they do in monogamous relationships (Rodrigues et al., 2016; Fairbrother et al., 2019), and report less preoccupation with constraining relationship forces (i.e., feeling obligation rather than desire toward a partner), which is associated with greater self-reported quality of life (Rodrigues et al., 2019). Stults (2018) also found that gay and bisexual men involved in multi-partner mating reported that the conflict resolution strategies of CNM improved their relationship satisfaction, communication, and trust. This suggests that CNM may improve, rather than dissolve, cooperation and well-being within certain populations – a feature that should be valued by those who fear how public acceptance of CNM might affect social cohesion.

### Limitations and Future Directions

The most notable limitation of this research is that it does not assess the influence of measured morality or sexual ethics on behavior within CNM relationships, and these are constructs that should be examined further in future work. Our results should not be interpreted as support for condemnation against CNM. Rather, our data highlight how those with a proclivity toward CNM may possess personality traits that predispose them to take risks, pursue multi-partner mating, and disregard pathogens. CNM may therefore not foster these traits, but rather provide an environment where people can ethically express them. Without strict ethical guidelines for how to handle multiple concurrent romantic relationships, people may pursue multi-partner mating in a manner that produces social disharmony. For example, in sub-Saharan and Muslim populations where polygamy is socially acceptable, women in polygamous relationships experience more spousal mistreatment, abuse, and mental health concerns than those in monogamous relationships (Hassouneh-Phillips, 2001; Özer et al., 2013). Children from these polygynous families also report more mental health and social difficulties, poorer school achievement, and poorer paternal relationships than those from monogamous families (Al-Krenawi et al., 2002; Al-Krenawi and Slonim-Nevo, 2008). Within these populations, these negative outcomes seem to arise when there is competition, hostility, jealousy, and little communication among partners. However, when effort is invested into building respectful and congenial relationships among partners, these outcomes improve (Al-Krenawi, 1998). This suggests that the dynamic of a multi-partner relationship may be a better predictor of relationship functioning than its structure (Elbedour et al., 2002). CNM ethical practices may likewise reduce conflict among those who pursue multi-partner relationships. Specifically, CNM’s culture of compassionately enforced sexual ethics may provide an outlet for fast life history strategists to pursue their preferred strategy in a manner that reduces its negative impact on others’ well-being.

This research highlights the need to identify and quantify a formal taxonomy of CNM ethics. Although a number of popular guides exist (e.g., Anapol, 1997; Hardy and Easton, 2017), there is no unified scientific examination of the diverse strategies that CNM practitioners use to manage multi-partner relationships. The most obvious ethical guideline that differentiates CNM from other forms of non-monogamy is its namesake: consent. However, this is too broad a concept to capture the myriad of ethical considerations that may arise within a multi-partner relationship. For example, Peoples et al. (2019) presented case studies of two married men who pursued extramarital partnerships with and without the consent of their spouse. They documented that both men, regardless of spouse consent, engaged in antagonistic and exploitative relationship practices, such as deception, partner neglect, and divestment from childcare, which subsequently produced relationship conflict. This suggests that consent-seeking is a nominal feature of CNM relationships and that ethical pursuit of multi-partner mating may instead require a multifaceted approach that addresses the diverse array of anxieties and exploitations that can produce suffering within romantic and interpersonal relationships.

It may be fruitful to begin this investigation by examining how CNM practices complement the recurrent, domain-specific adaptive issues that have shaped humans’ evolved psychology. Natural selection has shaped psychological adaptations that protect against cuckoldry and partner abandonment (Buss and Schmitt, 1993, 2019), interpersonal exploitation (Buss and Duntley, 2008; Duntley, 2015), and infection by disease (Al-Shawaf et al., 2015; Tybur and Lieberman, 2016). Although these adaptations may have enhanced reproductive success, they do not necessarily enhance well-being (Kováč, 2012), nor may they function optimally within a modern environment (Li et al., 2018). It is possible that the sexual ethics of CNM, paired with modern sexual health technologies, reduce the need for humans to rely on psychological mechanisms of disgust and moral condemnation to regulate sexual risk-taking and other features of a faster life history. For example, proscribing hostility among partners within CNM relationships may reduce intrasexual competition and its consequences on public health (see Kruger, 2010; Tybur et al., 2012). Future research should compare CNM individuals who adhere or not to the ethical principles espoused by the greater community and assess whether adherence tends to improve relationship functioning, particularly among those who have a predisposition to disregard others’ well-being.

Another limitation of this study is that it did not examine a complete array of life history traits. It also relies exclusively on self-report measures, which are vulnerable to revisionism and faulty memory. The validity of the Mini-K and HKSS as self-report measures of life history variation is contested (see Dunkel and Decker, 2010; Figueredo et al., 2013, 2015; see also Copping et al., 2014; Richardson et al., 2017), though our complementary measures provide convergent evidence that CNM is associated with a faster life history. Nevertheless, future research should examine a wider collection of behavioral measures of life history within CNM populations and consider which features of a fast life history are most endemic to CNM populations. Research should also address whether life history features are invariant across different CNM populations and subcultures (e.g., swinging vs. polyamory vs. religious polygamy). People within polyamorous relationships are typically viewed as more moral and responsible than those in swinging and open relationships (Matsick et al., 2014). To the extent that polyamorous relationships are defined by multiple close, emotionally intimate bonds, these relationships (and the people within them) may be seen as less socially disruptive. Similarly, we did not assess whether our participants had children, which can substantially shape relationship behaviors and attitudes (e.g., Barbaro et al., 2016; Flegr et al., 2019).

Finally, there are several methodological issues that should be considered when interpreting this data. First, several of our measures had low Cronbach’s alphas, including the MMSO and the ethical, health/safety, and social risk-taking facets of the DOSPERT. Similarly, our measure of pubertal development relied on self-report responses, which may be biased by retrospection. Research designs that rely on alternative, well-validated measures of psychological and social functioning (e.g., psychophysiological assessment; social relations modeling) administered within laboratory or naturalistic settings may help to improve the quality of life history and CNM research more broadly.

## Conclusion

Individuals in CNM relationships were more likely to report a fast life history than those in monogamous relationships. We speculate that this association may explain moral stigma toward CNM insofar as a faster life history is associated with risky, competitive, antagonistic interpersonal behaviors. Those who benefit from maintaining stable, cohesive groups may favor monogamy and condemn CNM to the extent that multi-partner mating can produce transient relationships, social conflict, and disease transmission; although, as noted, these traits do not necessarily describe individuals in modern CNM relationships. Given existing evidence that CNM relationships are not short-lived (Mogilski et al., 2017; Séguin et al., 2017), can improve relationship satisfaction and functioning (Rodrigues et al., 2016; Levine et al., 2018; Stults, 2018; Fairbrother et al., 2019), and are no more likely to involve unsafe sexual practices than monogamous relationships (Conley et al., 2012, 2013b; Lehmiller, 2015), we suspect that moral stigma toward CNM originates from an increasingly defunct intuitive association between sexually promiscuous mating and interpersonally deleterious fast life history traits (Moon et al., 2018). This mismatch (Li et al., 2018) may be driven by modern CNM ethical practices which reduce sources of interpersonal conflict within multi-partner mating systems (e.g., intrasexual competition, jealous anxiety, partner abandonment, child neglect, and disease transmission). Identifying which common CNM practices most effectively minimize these concerns will be the next step in this fruitful line of research.

## Data Availability Statement

The datasets generated for this study are available on request to the corresponding author.

## Ethics Statement

The studies involving human participants were reviewed and approved by the Oakland University Institutional Review Board. The patients/participants provided their written informed consent to participate in this study.

## Author Contributions

All authors contributed to the study design conception and collection of data. JM, VM, and SR organized and analyzed the data. JM wrote the manuscript. LW provided the editorial feedback.

## Conflict of Interest

The authors declare that the research was conducted in the absence of any commercial or financial relationships that could be construed as a potential conflict of interest.

## References

[B1] Aguilar J. (2013). Situational sexual behaviors: the ideological work of moving toward polyamory in communal living groups. *J. Contemp. Ethnogr.* 42 104–129. 10.1177/0891241612464886

[B2] Al-Krenawi A. (1998). Family therapy with a multiparental/multispousal family. *Fam. Process* 37 65–81. 10.1111/j.1545-5300.1998.00065.x 9589282

[B3] Al-Krenawi A. Graham J. R. Slonim-Nevo V. (2002). Mental health aspects of Arab-Israeli adolescents from polygamous versus monogamous families. *J. Soc. Psychol.* 142 446–460. 10.1080/00224540209603911 12153122

[B4] Al-Krenawi A. Slonim-Nevo V. (2008). Psychosocial and familial functioning of children from polygynous and monogamous families. *J. Soc. Psychol.* 148 745–764. 10.3200/SOCP.148.6.745-764 19058661

[B5] Al-Shawaf L. Lewis D. M. Buss D. M. (2015). Disgust and mating strategy. *Evol. Hum. Behav.* 36 199–205. 10.1016/j.appet.2014.10.029 25450899

[B6] Anapol D. (1997). *Polyamory: The New Love Without Limits.* San Rafael, CA: Internet Resource Center.

[B7] Ateah C. A. Snow W. Wener P. MacDonald L. Metge C. Davis P. (2011). Stereotyping as a barrier to collaboration: does interprofessional education make a difference? *Nurse Educ. Today* 31 208–213. 10.1016/j.nedt.2010.06.004 20655633

[B8] Balzarini R. N. Campbell L. Kohut T. Holmes B. M. Lehmiller J. J. Harman J. J. (2017). Perceptions of primary and secondary relationships in polyamory. *PLoS One* 12:e0177841. 10.1371/journal.pone.0177841 28542619 PMC5436896

[B9] Balzarini R. N. Dharma C. Kohut T. Campbell L. Lehmiller J. J. Harman J. J. (2019). Comparing relationship quality across different types of romantic partners in polyamorous and monogamous relationships. *Arch. Sex. Behav.* 48 1749–1767. 10.1007/s10508-019-1416-7 31069571

[B10] Balzarini R. N. Shumlich E. J. Kohut T. Campbell L. (2018a). Dimming the “halo” around monogamy: re-assessing stigma surrounding consensually non-monogamous romantic relationships as a function of personal relationship orientation. *Front. Psychol.* 9:894. 10.3389/fpsyg.2018.00894 30008682 PMC6034202

[B11] Balzarini R. N. Shumlich E. J. Kohut T. Campbell L. (2018b). Sexual attitudes, erotophobia, and sociosexual orientation differ based on relationship orientation. *J. Sex Res.* 10.1080/00224499.2018.1523360 [Epub ahead of print]. 30307752

[B12] Barbaro N. Shackelford T. K. Weekes-Shackelford V. A. (2016). Mothers and fathers perform more mate retention behaviors than individuals without children. *Hum. Nat.* 27 316–333. 10.1007/s12110-016-9261-z 27147537

[B13] Baumard N. Chevallier C. (2015). The nature and dynamics of world religions: a life-history approach. *Proc. R. Soc. B Biol. Sci.* 282:20151593. 10.1098/rspb.2015.1593 26511055 PMC4650154

[B14] Blais A. R. Weber E. U. (2006). A domain-specific risk-taking (DOSPERT) scale for adult populations. *Judgm. Decis. Mak.* 1 33–47.

[B15] Boothroyd L. G. Craig P. S. Crossman R. J. Perrett D. I. (2013). Father absence and age at first birth in a Western sample. *Am. J. Hum. Biol.* 25 366–369. 10.1002/ajhb.22378 23564358

[B16] Brandon M. (2016). Monogamy and nonmonogamy: evolutionary considerations and treatment challenges. *Sex. Med. Rev.* 4 343–352. 10.1016/j.sxmr.2016.05.005 27872028

[B17] Burleigh T. J. Rubel A. N. Meegan D. V. (2017). Wanting ‘the whole loaf’: zero-sum thinking about love is associated with prejudice against consensual non-monogamists. *Psychol. Sex.* 8 24–40. 10.1080/19419899.2016.1269020

[B18] Buss D. M. Duntley J. D. (2008). Adaptations for exploitation. *Group Dyn.* 12 53–62. 10.1037/1089-2699.12.1.53

[B19] Buss D. M. Schmitt D. P. (1993). Sexual strategies theory: an evolutionary perspective on human mating. *Psychol. Rev.* 100 204–232. 10.1037//0033-295x.100.2.204 8483982

[B20] Buss D. M. Schmitt D. P. (2019). Mate preferences and their behavioral manifestations. *Annu. Rev. Psychol.* 70 77–110. 10.1146/annurev-psych-010418-103408 30230999

[B21] Byrd-Craven J. Geary D. C. Vigil J. M. Hoard M. K. (2007). One mate or two? Life history traits and reproductive variation in low-income women. *Acta Psychol. Sin.* 39 469–480.

[B22] Cassidy T. Wong G. (2018). Consensually nonmonogamous clients and the impact of mononormativity in therapy. *Can. J. Couns. Psychother.* 52 119–139.

[B23] Conley T. D. Moors A. C. Matsick J. L. Ziegler A. (2013a). The fewer the merrier: assessing stigma surrounding consensually non-monogamous romantic relationships. *Anal. Soc. Issues Public Policy* 13 1–30. 10.1111/j.1530-2415.2012.01286.x

[B24] Conley T. D. Moors A. C. Ziegler A. Matsick J. L. Rubin J. D. (2013b). Condom use errors among sexually unfaithful and consensually nonmonogamous individuals. *Sex. Health* 10 463–464. 10.1071/SH12194 23726742

[B25] Conley T. D. Ziegler A. Moors A. C. (2013c). Backlash from the bedroom: stigma mediates gender differences in acceptance of casual sex offers. *Psychol. Women Q.* 37 392–407. 10.1177/0361684312467169

[B26] Conley T. D. Ziegler A. Moors A. C. Matsick J. L. Valentine B. (2013d). A critical examination of popular assumptions about the benefits and outcomes of monogamous relationships. *Pers. Soc. Psychol. Rev.* 17 124–141. 10.1177/1088868312467087 23175520

[B27] Conley T. D. Moors A. C. Ziegler A. Karathanasis C. (2012). Unfaithful individuals are less likely to practice safer sex than openly nonmonogamous individuals. *J. Sex. Med.* 9 1559–1565. 10.1111/j.1743-6109.2012.02712.x 22463058

[B28] CoppingL. T.CampbellA.MuncerS. (2014). Psychometrics and life history strategy: the structure and validity of the high K strategy scale. *Evol. Psychol.* 12 200–222. 25299760

[B29] Day M. V. (2013). Stigma, halo effects, and threats to ideology: comment on the fewer the merrier? *Anal. Soc. Issues Public Policy* 13 49–51. 10.1111/asap.12005

[B30] Day M. V. Kay A. C. Holmes J. G. Napier J. L. (2011). System justification and the defense of committed relationship ideology. *J. Pers. Soc. Psychol.* 101 291–306. 10.1037/a0023197 21480736

[B31] Del Giudice M. Gangestad S. W. Kaplan H. S. (2015). “Life history theory and evolutionary psychology,” in *The Handbook of Evolutionary Psychology, Foundations*, Vol. 1 ed. Buss D. M. (Hoboken, NJ: Jon Wiley & Sons), 88–114.

[B32] Deri J. (2015). *Love’s Refraction: Jealousy and Compersion in Queer Women’s Polyamorous Relationships.* Toronto: University of Toronto Press.

[B33] DeScioli P. Kurzban R. (2009). Mysteries of morality. *Cognition* 112 281–299. 10.1016/j.cognition.2009.05.008 19505683

[B34] DeScioli P. Kurzban R. (2013). A solution to the mysteries of morality. *Psychol. Bull.* 139 477–496. 10.1037/a0029065 22747563

[B35] Duncan L. A. Schaller M. Park J. H. (2009). Perceived vulnerability to disease: development and validation of a 15-item self-report instrument. *Pers. Individ. Differ.* 47 541–546. 10.1016/j.paid.2009.05.001

[B36] Dunkel C. S. Decker M. (2010). Convergent validity of measures of life-history strategy. *Pers. Individ. Differ.* 48 681–684. 10.1080/10401334.2017.1414608 29351403

[B37] Dunkel C. S. Summerville L. A. Mathes E. W. Kesserling S. N. (2015). Using the California Q-sort measure of life history strategy to predict sexual behavioral outcomes. *Arch. Sex. Behav.* 44 1705–1711. 10.1007/s10508-014-0445-5 25515585

[B38] Duntley J. D. (2015). “Adaptations to dangers from humans,” in *The Handbook of Evolutionary Psychology*, ed. Buss D. M. (Hoboken, NJ: John Wiley & Sons), 264–286.

[B39] Elbedour S. Onwuegbuzie A. J. Caridine C. Abu-Saad H. (2002). The effect of polygamous marital structure on behavioral, emotional, and academic adjustment in children: a comprehensive review of the literature. *Clin. Child Fam. Psychol. Rev.* 5 255–271. 12495269 10.1023/a:1020925123016

[B40] Ellis B. J. Del Giudice M. (2019). Developmental adaptation to stress: an evolutionary perspective. *Annu. Rev. Psychol.* 70 111–139. 10.1146/annurev-psych-122216-011732 30125133

[B41] Emens E. F. (2004). Monogamy’s law: compulsory monogamy and polyamorous existence. *N. Y. Univ. Rev. L. Soc. Change* 29 277–376.

[B42] Fairbrother N. Hart T. A. Fairbrother M. (2019). Open relationship prevalence, characteristics, and correlates in a nationally representative sample of Canadian adults. *J. Sex Res.* 56 695–704. 10.1080/00224499.2019.1580667 30932711

[B43] Figueredo A. J. de Baca T. C. Black C. J. García R. A. Fernandes H. B. F. Wolf P. S. A. (2015). Methodologically sound: evaluating the psychometric approach to the assessment of human life history [reply to Copping, Campbell, and Muncer, 2014]. *Evol. Psychol.* 13 299–338. 25844774 10.1177/147470491501300202 PMC4845720

[B44] Figueredo A. J. de Baca T. C. Woodley M. A. (2013). The measurement of human life history strategy. *Pers. Individ. Differ.* 55 251–255. 10.1016/j.paid.2012.04.033

[B45] Figueredo A. J. Jacobs W. J. (2010). “Aggression, risk taking, and alternative life history strategies: the behavioral ecology of social deviance,” in *Biopsycho-Social Perspectives on Interpersonal Violence*, eds Frias-Armenta M. Corral-Verdugo V. (Hauppauge, NY: NOVA Science Publishers), 3–28.

[B46] Figueredo A. J. Jacobs W. J. Gladden P. R. Bianchi J. Patch E. A. Kavanagh P. S. (2018). Intimate partner violence, interpersonal aggression, and life history strategy. *Evol. Behav. Sci.* 12 1–31.

[B47] Figueredo A. J. Vásquez G. Brumbach B. H. Schneider S. M. R. Sefcek J. A. Tal I. R. (2006). Consilience and life history theory: from genes to brain to reproductive strategy. *Dev. Rev.* 26 243–275. 10.1016/j.dr.2006.02.002

[B48] Finn M. D. Tunariu A. D. Lee K. C. (2012). A critical analysis of affirmative therapeutic engagements with consensual non-monogamy. *Sex. Relation. Ther.* 27 205–216. 10.1080/14681994.2012.702893

[B49] Flegr J. Kroupa S. Nekola O. Blum A. E. (2019). What people prefer and what they think they prefer in short- and long-term partners. The effects of the phase of the menstrual cycle, hormonal contraception, pregnancy, and the marital and the parenthood status on partner preferences. *Evol. Hum. Behav.* 40 112–125. 10.1016/j.evolhumbehav.2018.09.003

[B50] Frankenhuis W. E. Panchanathan K. Nettle D. (2016). Cognition in harsh and unpredictable environments. *Curr. Opin. Psychol.* 7 76–80. 10.1007/s10071-011-0425-2 21670948 PMC3645319

[B51] Frederick M. J. Keil H. R. Bassioni R. Khan H. (2018). Higher scores on the Mini-K life history battery are associated with greater disgust sensitivity on the three domains of disgust scale. *Evol. Mind Behav.* 16 37–51. 10.1556/2050.2018.0002

[B52] Gangestad S. W. Simpson J. A. (2000). The evolution of human mating: trade-offs and strategic pluralism. *Behav. Brain Sci.* 23 573–587. 10.1017/s0140525x0000337x 11301543

[B53] Giosan C. (2006). High-K strategy scale: a measure of the high-K independent criterion of fitness. *Evol. Psychol.* 4 394–405.

[B54] Griskevicius V. Tybur J. M. Delton A. W. Robertson T. E. (2011). The influence of mortality and socioeconomic status on risk and delayed rewards: a life history theory approach. *J. Pers. Soc. Psychol.* 100 1015–1026. 10.1037/a0022403 21299312 PMC3298774

[B55] Grunt-Mejer K. Campbell C. (2016). Around consensual nonmonogamies: assessing attitudes toward nonexclusive relationships. *J. Sex Res.* 53 45–53. 10.1080/00224499.2015.1010193 26241075

[B56] Hackman J. Hruschka D. (2013). Fast life histories, not pathogens, account for state-level variation in homicide, child maltreatment, and family ties in the US. *Evol. Hum. Behav.* 34 118–124. 10.1016/j.evolhumbehav.2012.11.002

[B57] Hampson S. E. Andrews J. A. Barckley M. Gerrard M. Gibbons F. X. (2016). Harsh environments, life history strategies, and adjustment: a longitudinal study of Oregon youth. *Pers. Individ. Differ.* 88 120–124. 10.1016/j.paid.2015.08.052 26451065 PMC4593070

[B58] Hardy J. W. Easton D. (2017). *The Ethical Slut: A Practical Guide to Polyamory, Open Relationships, and other Adventures*, 3rd Edn New York, NY: Penguin Random House.

[B59] Hassouneh-Phillips D. (2001). Polygamy and wife abuse: a qualitative study of Muslim women in America. *Health Care Women Int.* 22 735–748. 10.1080/073993301753339951 11813788

[B60] Hehman J. A. Salmon C. A. (2019). Sex-specific developmental effects of father absence on casual sexual behavior and life history strategy. *Evol. Psychol. Sci.* 5 121–130. 10.1007/s40806-018-0173-5

[B61] Hill M. E. Augoustinos M. (2001). Stereotype change and prejudice reduction: short-and long-term evaluation of a cross–cultural awareness programme. *J. Commun. Appl. Soc. Psychol.* 11 243–262. 10.1002/casp.629.abs

[B62] Hogan D. E. Mallott M. (2005). Changing racial prejudice through diversity education. *J. Coll. Stud. Dev.* 46 115–125. 10.1353/csd.2005.0015

[B63] Hutzler K. T. Giuliano T. A. Herselman J. R. Johnson S. M. (2016). Three’s a crowd: public awareness and (mis) perceptions of polyamory. *Psychol. Sex.* 7 69–87. 10.1080/19419899.2015.1004102

[B64] Jackson J. J. Kirkpatrick L. A. (2007). The structure and measurement of human mating strategies: toward a multidimensional model of sociosexuality. *Evol. Hum. Behav.* 28 382–391. 10.1016/j.evolhumbehav.2007.04.005

[B65] Jackson S. Scott S. (2004). The personal is still political: heterosexuality, feminism and monogamy. *Fem. Psychol.* 14 151–157. 10.1177/0959353504040317

[B66] James J. Ellis B. J. Schlomer G. L. Garber J. (2012). Sex-specific pathways to early puberty, sexual debut, and sexual risk taking: tests of an integrated evolutionary–developmental model. *Dev. Psychol.* 48 687–702. 10.1037/a0026427 22268605

[B67] Kirkman L. Dickson-Swift V. Fox C. (2015). Midlife relationship diversity, sexual fluidity, wellbeing and sexual health from a rural perspective. *Rural Soc.* 24 266–281. 10.1080/10371656.2015.1099272

[B68] Klesse C. (2018). Theorizing multi-partner relationships and sexualities–Recent work on non-monogamy and polyamory. *Sexualities* 21 1109–1124. 10.1177/1363460717701691

[B69] Kováč L. (2012). The biology of happiness: chasing pleasure and human destiny. *EMBO Rep.* 13 297–302. 22410831 10.1038/embor.2012.26 PMC3321158

[B70] KrugerD. J. (2010). Socio-demographic factors intensifying male mating competition exacerbate male mortality rates. *Evol. Psychol.* 8 194–204. 22947791

[B71] Kulik C. T. Roberson L. (2008). Common goals and golden opportunities: evaluations of diversity education in academic and organizational settings. *Acad. Manag. Learn. Educ.* 7 309–331. 10.5465/amle.2008.34251670

[B72] Kurzban R. Dukes A. Weeden J. (2010). Sex, drugs and moral goals: reproductive strategies and views about recreational drugs. *Proc. R. Soc. B Biol. Sci.* 277 3501–3508. 10.1098/rspb.2010.0608 20554547 PMC2982222

[B73] Kuzawa C. W. Bragg J. M. (2012). Plasticity in human life history strategy: implications for contemporary human variation and the evolution of genus Homo. *Curr. Anthropol.* 53 S369–S382.

[B74] Lehmiller J. J. (2015). A comparison of sexual health history and practices among monogamous and consensually nonmonogamous sexual partners. *J. Sex. Med.* 12 2022–2028. 10.1111/jsm.12987 26395880

[B75] Levine E. C. Herbenick D. Martinez O. Fu T. C. Dodge B. (2018). Open relationships, nonconsensual nonmonogamy, and monogamy among US adults: findings from the 2012 National Survey of Sexual Health and Behavior. *Arch. Sex. Behav.* 47 1439–1450. 10.1007/s10508-018-1178-7 29696552 PMC5958351

[B76] Li N. P. van Vugt M. Colarelli S. M. (2018). The evolutionary mismatch hypothesis: implications for psychological science. *Curr. Dir. Psychol. Sci.* 27 38–44. 10.1177/0963721417731378

[B77] Lukaszewski A. W. (2015). Parental support during childhood predicts life history-related personality variation and social status in young adults. *Evol. Psychol. Sci.* 1 131–140. 10.1007/s40806-015-0015-7

[B78] Maner J. K. Dittmann A. Meltzer A. L. McNulty J. K. (2017). Implications of life-history strategies for obesity. *Proc. Natl. Acad. Sci. U.S.A.* 114 8517–8522. 10.1073/pnas.1620482114 28739939 PMC5558992

[B79] Manley M. H. Diamond L. M. van Anders S. M. (2015). Polyamory, monoamory, and sexual fluidity: a longitudinal study of identity and sexual trajectories. *Psychol. Sex. Orientat. Gend. Divers.* 2 168–180. 10.1037/sgd0000098

[B80] Matsick J. L. Conley T. D. Ziegler A. Moors A. C. Rubin J. D. (2014). Love and sex: polyamorous relationships are perceived more favourably than swinging and open relationships. *Psychol. Sex.* 5 339–348. 10.1080/19419899.2013.832934

[B81] McDonald M. M. Donnellan M. B. Navarrete C. D. (2012). A life history approach to understanding the Dark Triad. *Pers. Individ. Differ.* 52 601–605. 10.1016/j.paid.2011.12.003

[B82] McLean K. (2004). Negotiating (non) monogamy: bisexuality and intimate relationships. *J. Bisex.* 4 83–97. 10.1300/j159v04n01_07

[B83] Međedović J. (2018). Exploring the links between psychopathy and life history in a sample of college females: a behavioral ecological approach. *Evol. Psychol. Sci.* 4 466–473. 10.1007/s40806-018-0157-5

[B84] Minkov M. Beaver K. (2016). A test of life history strategy theory as a predictor of criminal violence across 51 nations. *Pers. Individ. Differ.* 97 186–192. 10.1016/j.paid.2016.03.063

[B85] Mishra S. Templeton A. J. Meadows T. J. (2017). Living, fast and slow: is life history orientation associated with risk-related personality traits, risk attitudes, criminal outcomes, and gambling? *Pers. Individ. Differ.* 117 242–248. 10.1016/j.paid.2017.06.009

[B86] Mitchell M. E. Bartholomew K. Cobb R. J. (2014). Need fulfillment in polyamorous relationships. *J. Sex Res.* 51 329–339. 10.1080/00224499.2012.742998 23541166

[B87] Mogilski J. K. Memering S. L. Welling L. L. Shackelford T. K. (2017). Monogamy versus consensual non-monogamy: alternative approaches to pursuing a strategically pluralistic mating strategy. *Arch. Sex. Behav.* 46 407–417. 10.1007/s10508-015-0658-2 26679305

[B88] Mogilski J. K. Reeve S. D. Nicolas S. C. Donaldson S. H. Mitchell V. E. Welling L. L. (2019). Jealousy, consent, and compersion within monogamous and consensually non-monogamous romantic relationships. *Arch. Sex. Behav.* 48 1811–1828. 10.1007/s10508-018-1286-4 30607710

[B89] Moon J. W. Krems J. A. Cohen A. B. (2018). Religious people are trusted because they are viewed as slow life-history strategists. *Psychol. Sci.* 29 947–960. 10.1177/0956797617753606 29590005

[B90] Moors A. C. (2019). Moving past the rose-tinted lens of monogamy: onward with critical self-examination and (sexually) healthy science. *Arch. Sex. Behav.* 48 57–61. 10.1007/s10508-018-1215-6 29663164

[B91] Moors A. C. Matsick J. L. Schechinger H. A. (2017). Unique and shared relationship benefits of consensually non-monogamous and monogamous relationships. *Eur. Psychol.* 22 55–71. 10.1027/1016-9040/a000278

[B92] Moors A. C. Matsick J. L. Ziegler A. Rubin J. D. Conley T. D. (2013). Stigma toward individuals engaged in consensual nonmonogamy: robust and worthy of additional research. *Anal. Soc. Issues Public Policy* 13 52–69. 10.1111/asap.12020

[B93] Morrison T. G. Beaulieu D. Brockman M. Beaglaoich C. Ó. (2013). A comparison of polyamorous and monoamorous persons: are there differences in indices of relationship well-being and sociosexuality? *Psychol. Sex.* 4 75–91. 10.1080/19419899.2011.631571

[B94] Muise A. Laughton A. K. Moors A. Impett E. A. (2019). Sexual need fulfillment and satisfaction in consensually nonmonogamous relationships. *J. Soc. Pers. Relatsh.* 36 1917–1938. 10.1177/0265407518774638

[B95] Nettle D. Colléony A. Cockerill M. (2011). Variation in cooperative behaviour within a single city. *PLoS One* 6:e26922. 10.1371/journal.pone.0026922 22046411 PMC3203179

[B96] Nettle D. Frankenhuis W. E. (2019). The evolution of life-history theory: a bibliometric analysis of an interdisciplinary research area. *Proc. R. Soc. B Biol. Sci.* 286:20190040. 10.1098/rspb.2019.0040 30914012 PMC6452081

[B97] Özer A. Orhan F. Ö. Ekerbiçer H. Ç. (2013). Sociodemographic variables and depression in Turkish women from polygamous versus monogamous families. *Health Care Women Int.* 34 1024–1034. 10.1080/07399332.2012.692414 23445340

[B98] Peoples K. Zazzarino A. Thompson J. Riley P. Shelton D. Fisher R. (2019). The unfaithful male in monogamous and non-monogamous marriage: a phenomenological case study. *Sex. Addict. Compulsivity* 26 137–163. 10.1080/10720162.2019.1608880

[B99] Pepper G. V. Nettle D. (2017). The behavioural constellation of deprivation: causes and consequences. *Behav. Brain Sci.* 40:e314. 10.1017/S0140525X1600234X 28073390

[B100] Petersen A. C. Crockett L. Richards M. Boxer A. (1988). A self-report measure of pubertal status: reliability, validity, and initial norms. *J. Youth Adolesc.* 17 117–133. 10.1007/BF01537962 24277579

[B101] Richardson G. B. Chen C. C. Dai C. L. Brubaker M. D. Nedelec J. L. (2017). The psychometrics of the Mini-K: evidence from two college samples. *Evol. Psychol.* 15:1474704916682034. 10.1177/1474704916682034 PMC10996849 28152621

[B102] RichardsonG. B.ChenC. C.DaiC. L.HardestyP. H.SwobodaC. M. (2014). Life history strategy and young adult substance use. *Evol. Psychol.* 12 932–957. 25365695

[B103] Rodrigues D. Fasoli F. Huic A. Lopes D. (2018). Which partners are more human? Monogamy matters more than sexual orientation for dehumanization in three European countries. *Sex. Res. Soc. Policy* 15 504–515. 10.1007/s13178-017-0290-0

[B104] Rodrigues D. Lopes D. Pereira M. (2016). “We agree and now everything goes my way”: consensual sexual nonmonogamy, extradyadic sex, and relationship satisfaction. *Cyberpsychol. Behav. Soc. Netw.* 19 373–379. 10.1089/cyber.2016.0114 27327064

[B105] Rodrigues D. Lopes D. Smith C. V. (2017). Caught in a “bad romance”? Reconsidering the negative association between sociosexuality and relationship functioning. *J. Sex Res.* 54 1118–1127. 10.1080/00224499.2016.1252308 27911084

[B106] Rodrigues D. L. Lopes D. Pereira M. De Visser R. Cabaceira I. (2019). Sociosexual attitudes and quality of life in (non) monogamous relationships: the role of attraction and constraining forces among users of the Second Love web site. *Arch. Sex. Behav.* 48 1795–1809. 10.1007/s10508-018-1272-x 30607714

[B107] Rubel A. N. Bogaert A. F. (2015). Consensual nonmonogamy: psychological well-being and relationship quality correlates. *J. Sex Res.* 52 961–982. 10.1080/00224499.2014.942722 25189189

[B108] Salmon C. Townsend J. M. Hehman J. (2016). Casual sex and college students: sex differences and the impact of father absence. *Evol. Psychol. Sci.* 2 254–261. 10.1007/s40806-016-0061-9

[B109] Salvatore J. (2013). Back to basics in the assessment of stigma: commentary on Conley et al.(2012). *Anal. Soc. Issues Public Policy* 13 45–48. 10.1111/asap.12003

[B110] Schechinger H. A. Sakaluk J. K. Moors A. C. (2018). Harmful and helpful therapy practices with consensually non-monogamous clients: toward an inclusive framework. *J. Consult. Clin. Psychol.* 86 879–891. 10.1037/ccp0000349 30335421

[B111] Scoats R. Anderson E. (2019). ‘My partner was just all over her’: jealousy, communication and rules in mixed-sex threesomes. *Cult. Health Sex.* 21 134–146. 10.1080/13691058.2018.1453088 30764748

[B112] Sear R. Sheppard P. Coall D. A. (2019). Cross-cultural evidence does not support universal acceleration of puberty in father-absent households. *Philos. Trans. R. Soc. B Biol. Sci.* 374:20180124. 10.1098/rstb.2018.0124 30966893 PMC6460089

[B113] Séguin L. J. Blais M. Goyer M. F. Adam B. D. Lavoie F. Rodrigue C. (2017). Examining relationship quality across three types of relationship agreements. *Sexualities* 20 86–104. 10.1177/1363460716649337

[B114] Stults C. B. (2018). Relationship quality among young gay and bisexual men in consensual nonmonogamous relationships. *J. Soc. Pers. Relat.* 36:026540751880953.

[B115] Thompson A. E. Bagley A. J. Moore E. A. (2018). Young men and women’s implicit attitudes towards consensually nonmonogamous relationships. *Psychol. Sex.* 9 117–131. 10.1080/19419899.2018.1435560

[B116] TyburJ. M.BryanA. D.HooperA. E. C. (2012). An evolutionary perspective on health psychology: new approaches and applications. *Evol. Psychol.* 10 855–867. 23253791

[B117] Tybur J. M. Lieberman D. (2016). Human pathogen avoidance adaptations. *Curr. Opin. Psychol.* 7 6–11. 10.1016/j.copsyc.2015.06.005

[B118] Tybur J. M. Lieberman D. Kurzban R. DeScioli P. (2013). Disgust: evolved function and structure. *Psychol. Rev.* 120 65–84. 10.1037/a0030778 23205888

[B119] van Tol R. (2017). I love you, and you, and you too: challenges of consensual nonmonogamy in relationship therapy. *Trans. Anal. J.* 47 276–293. 10.1177/0362153717720191

[B120] Visser R. McDonald D. (2007). Swings and roundabouts: management of jealousy in heterosexual ‘swinging’ couples. *Br. J. Soc. Psychol.* 46 459–476. 10.1348/014466606x143153 17565792

[B121] Wang X. T. Kruger D. J. Wilke A. (2009). Life history variables and risk-taking propensity. *Evol. Hum. Behav.* 30 77–84. 10.1016/j.evolhumbehav.2008.09.006

[B122] Weeden J. Cohen A. B. Kenrick D. T. (2008). Religious attendance as reproductive support. *Evol. Hum. Behav.* 29 327–334. 10.1016/j.evolhumbehav.2008.03.004 21874105 PMC3161130

[B123] Williams K. E. Sng O. Neuberg S. L. (2016). Ecology-driven stereotypes override race stereotypes. *Proc. Natl. Acad. Sci. U.S.A.* 113 310–315. 10.1073/pnas.1519401113 26712013 PMC4720338

[B124] Wosick-Correa K. (2010). Agreements, rules and agentic fidelity in polyamorous relationships. *Psychol. Sex.* 1 44–61. 10.1080/19419891003634471

[B125] Young E. S. Griskevicius V. Simpson J. A. Waters T. E. Mittal C. (2018). Can an unpredictable childhood environment enhance working memory? Testing the sensitized-specialization hypothesis. *J. Pers. Soc. Psychol.* 114 891–908. 10.1037/pspi0000124 29389153

[B126] Ziegler A. Matsick J. L. Moors A. C. Rubin J. Conley T. D. (2014). Does monogamy harm women? Deconstructing monogamy with a feminist lens. *J. Psychol.* 22 1–18.

[B127] Zietsch B. P. Sidari M. J. (2019). A critique of life history approaches to human trait covariation. *Evol. Hum. Behav.* (in press).

